# Effects of Iliosacral Joint Immobilization on Walking after Iliosacral Screw Fixation in Humans

**DOI:** 10.3390/jcm12206470

**Published:** 2023-10-11

**Authors:** Katharina Jäckle, Takashi Yoshida, Kira Neigefink, Marc-Pascal Meier, Mark-Tilmann Seitz, Thelonius Hawellek, Gabriela von Lewinski, Paul Jonathan Roch, Lukas Weiser, Arndt F. Schilling, Wolfgang Lehmann

**Affiliations:** Department of Trauma Surgery, Orthopaedics and Plastic Surgery, University Medical Center Göttingen, Robert-Koch Str. 40, 37075 Göttingen, Germany; yoshida.takashi@med.uni-goettingen.de (T.Y.); k.neigefink@med.uni-goettingen.de (K.N.); marc-pascal.meier@med.uni-goettingen.de (M.-P.M.); mark-tilmann.seitz@med.uni-goettingen.de (M.-T.S.); thelonius.hawellek@med.uni-goettingen.de (T.H.); gabriela.lewinski@med.uni-goettingen.de (G.v.L.); jonathan.roch@med.uni-goettingen.de (P.J.R.); lukas.weiser@med.uni-goettingen.de (L.W.); wolfgang.lehmann@med.uni-goettingen.de (W.L.)

**Keywords:** gait analysis, pelvic ring fracture, iliosacral joint immobilization, percutaneous screw fixation

## Abstract

Background: Pelvis fractures are commonly stabilized by surgical implants to facilitate their healing. However, such implants immobilize the iliosacral joint for up to a year until removal. We report how iliosacral joint immobilization affects the walking of patients. Methods: The gaits of patients with immobilized sacroiliac joints after unstable pelvic fracture (*n* = 8; mean age: 45.63 ± 23.19; five females and three males) and sex- and age-matched healthy control individuals (*n* = 8; mean age: 46.50 ± 22.91; five females and three males) were recorded and analyzed using a motion capture system. The forces between the tread and feet were also recorded. Standard gait parameters as well as dynamic patterns of joint angles and moments of the lower extremities were analyzed using the simulation software OpenSim. Results: With the exception of hip extensor strength, the monitored joint parameters of the patients showed task-dependent deviations during walking, i.e., plantarflexor force was increased when stepping on an elevated surface, as were hip flexion and extensor moments, knee flexion and extensor moments, as well as ankle dorsiflexion and the associated negative plantarflexor force during stance on the elevated surface. Conclusions: Iliosacral joint fixation causes reduced forward and upward propulsion and requires an extended range of hip motion in the sagittal plane. Patients show significant mobility limitation after iliosacral screw fixation.

## 1. Introduction

The pelvis is essential for mechanical stability and movement of the human body. It represents a ring formed by the bilateral hip bones (ilia, ischia, and pubes) and the sacrum (the triangular bone between the ilia). When the sacrum is fractured, for example as a result of high-energy trauma such as a motor vehicle accident [1,2], the pelvic ring is disrupted and thus no longer stable against rotational and vertical shear forces [3,4]. Conventionally, sacral fractures that lead to an unstable pelvic ring are stabilized by surgical implants, i.e., internal fixation. However, these implants also immobilize the joint between the ilium and sacrum (iliosacral joint) [1,5,6] for up to a year until the fracture has healed and the implant can be removed. Previous studies have not yet investigated how such sub-chronic immobilization of the iliosacral joint affects patient movement. Here, we report how surgical treatment by iliosacral joint screw immobilization affects the bipedal walking of patients. It is intuitive that the iliosacral joint plays a role in bipedal walking since it transmits force between the lower extremities and upper body [7]. As described by Lee [8], this joint enables relative rotation between the hip bones and sacrum during the gait cycle, i.e., the period between consecutive heel contact by the same foot. On the side of the swinging leg, the hip bone rotates posteriorly in the sagittal plane relative to the sacrum [8]. On the side of the stance leg, the hip bone rotates anteriorly in the sagittal plane relative to the sacrum [8]. These rotations have been observed during hip flexion and extension in the supine position, which are analogous to the swing and stance of the gait cycle [9], respectively. Eliminating such rotations by joint immobilization probably alters the gaits of patients. In fact, it is already known that very limited mobility and movement are present in the iliosacral joint during well controlled leg movements. However, whether this affects gait, during which muscle activity is much higher and could further stiffen the iliosacral joint, is still unknown and was also investigated as part of our study.

The effects of iliosacral joint immobilization can be inferred from gait impairments that occur after internal fixation of an unstable pelvic ring disruption, assuming that the observed individuals have undergone conventional methods of stabilization [1]. A few studies have reported that individuals with internally fixed pelvic ring disruptions exhibit a smaller average stride length with greater inter-individual variability compared to normative data [3,4]. Other studies have reported the mere presence of gait impairments [10] or simple descriptions based on visual inspection [11].

We aimed to gain deeper insight into the impact of current surgical treatment of unstable pelvic fractures on patient walking and to demonstrate how the iliosacral joint contributes to the normal gait of untreated individuals. Here, we report the differences in gait behavior of subjects with and without immobilization of the iliosacral joint. The results suggest that patients have limitations in their mobility after iliosacral screw fixation.

## 2. Materials and Methods

### 2.1. Patient Collective for Study

The present study was approved by the ethics committee of the University Medical Center Göttingen (approval number: AN 16/7/18) and was performed in compliance with the Helsinki Declaration.

The inclusion criteria for this prospective clinical study were defined by a patient collective at a university clinic in the period July 2016–May 2022, aged between 18 and 70 years, who were treated unilaterally at a university institution for a pelvic ring fracture of the iliosacral joint by percutaneous screw fixation (*n* = 8; mean age: 45.63 ± 23.19; five females and three males). The control individuals had not undergone surgical fixation (*n* = 8; mean age: 46.50 ± 22.91; five females and three males), had a physiological gait pattern, and were aged between 18 and 70 years.

The exclusion criteria were defined as follows:

(a) Medical conditions that may interfere with walking or impair gait (for example, significant soft tissue injury, infection, or pain; diagnosed movement disorder, such as Parkinson’s disease, essential tremor, or dystonia; stroke; and moderate to severe traumatic brain injury) or major limb amputation;
(b) Diagnosed dementia;
(c) Pregnancy;
(d) Diagnosed substance dependence;
(e) Inability to communicate with investigators;
(f) Uncooperative behavior;
(g) Inability to provide written informed consent;
(h) Severe comorbidities that made participation hazardous (e.g., severely reduced bone mineral density).

### 2.2. Gait Laboratory

The gait laboratory of the Applied Rehabilitation Technology Lab (ART-Lab) of the University Medical Center Göttingen was used to perform the experiments. The gait lab has a 12.5 m long test track with two force plates integrated to record the ground reaction forces. Motion data were collected using eight infrared cameras distributed throughout the room. A digital camera was used to record reference videos. All measurement systems could be started synchronously via a trigger.

### 2.3. Selection of Markers

Retroreflective markers from Qualisys (Qualisys AB, Goteborg, Sweden) were used for the experiments. The placement of the markers was based on the recommendations of the OpenSim user manual [12] and previous experiments. Markers were placed in such a way that they were affected as little as possible by skin, muscle, or soft tissue movement in order to avoid measurement artifacts. Anatomical markers were placed on defined and palpable bone points. They were later used to scale the model. Each body segment (trunk, pelvis, thigh, lower leg, foot) was defined by at least 3 markers to capture all degrees of freedom in three-dimensional space. To ensure this, three cluster markers each were placed in a triangle on the upper and lower leg in addition to the anatomical markers. One cluster tracker was placed on each of the upper and lower arm. The positions of the individual markers were not allowed to be too close to one another, otherwise the camera system could not distinguish them. On body segments with limited space, such as the feet and pelvis, the anatomical markers were also used as cluster markers.

### 2.4. Gait Analysis

**Figure 1:**
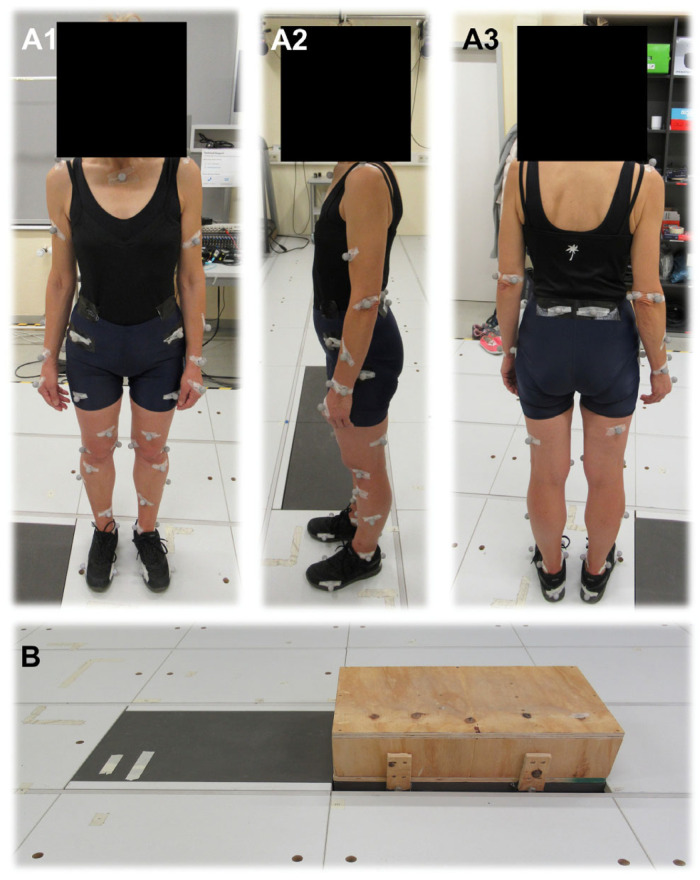
Design of measurements. Proband with retroreflective markers from Qualisys (Qualisys AB, Goteborg, Sweden) in front view (**A1**), side view (**A2**), and back view (**A3**). Step constructed of wood accurately fit on the force plate (**B**).

The gait analysis was performed according to a standardized study protocol and consisted of two tasks to be completed: “walking on a flat surface” and “crossing a step while walking on a flat surface”. The step was constructed of wood and fit exactly on the surface of the force plate (see Figure 1). The force acting on the step was thus transferred 1:1 to the plate. The step height was set at 17.5 cm (in accordance with building regulations in force in Germany and in accordance with TÜVSÜD guidelines) with orientation to the standard value. The order of the tasks was randomized for each subject to minimize external influences. During the gait tests, the subjects wore broken-in flat shoes that they had brought themselves.

### 2.5. Preparation of the Gait Laboratory

Preparation of the gait laboratory included calibration of the camera system and force plates and placement of the reference camera. The functions of all laboratory apparatus were checked. After collecting the subject’s data, their clothing and footwear were checked and adjusted. Clothing was tight fitting to minimize movement of the markers in relation to the skin. In addition, as few marker points as possible were covered by clothing. Footwear was comfortable for the subject and did not affect their gait. According to the marker protocol, the markers were placed on the subject with double-sided tape and fixed with surgical tape. To document the final placement of the markers, the subject was photographed in the anatomic position from all sides with a digital camera (Canon, PowerShot SX620 HS). Subsequently, the height and weight of the participants were measured to allow accurate interpretation of the measured kinematic data (see Table 1). Before starting the experiments, it was verified that all markers could be detected by the infrared cameras. In addition, the subjects were given a few minutes to get used to walking with the markers.

### 2.6. Static Measurement

Prior to performing the dynamic gait test, two static measurements were performed. The subject was asked to stand quietly in anatomical neutral-zero position with one foot on the force plate for a recording duration of two seconds. Using the captured experimental markers, the software was able to create a virtual model, define the position, and orient the reference cluster of each segment, and the movement recordings were later described relative to the virtual model. The static measurements had to be repeated during the experimental protocol in case a marker moved from its position.

### 2.7. Dynamic Measurement

The dynamic measurement consisted of the two tasks: “walking on a level surface” and “crossing a step while walking on a level surface”. Depending on the experimental condition, the position of the second force plate had to be recalibrated for the data acquisition software, i.e., adjusted to level ground or the height of the step. Basically, in both tasks, the subject walked along the test track at a self-selected speed. The subject’s movements were recorded using motion capture and the ground reaction forces were recorded using the force plates. The start of each measurement was signaled to the test person by a start signal. Simultaneously with the start signal, the measurement systems were activated synchronously by a manual trigger for a fixed period of time.

**Figure 2:**
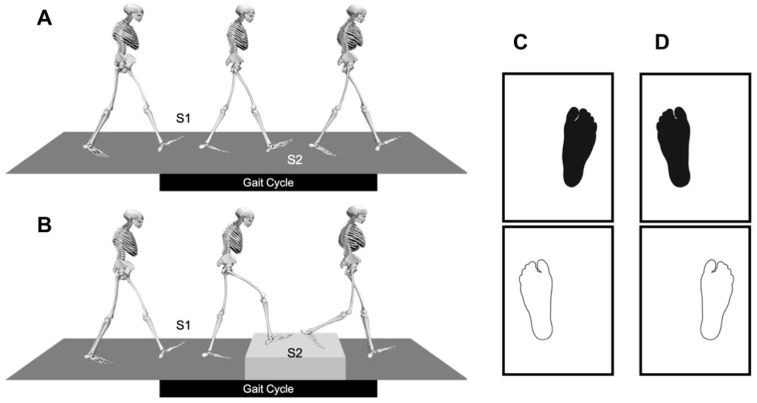
Experimental tasks: level-ground walking (**A**) and stepping onto and down from an elevated surface (height: 17.5 cm) while walking in a straight line on level ground (**B**). The time window of analysis (a gait cycle, defined by two consecutive foot contacts on the same side) containing two stances of interest (**S1** and **S2**). Correct foot placement (**C**,**D**). The left column (**C**) shows a pattern of correct foot placement: the left foot is positioned on the first force plate and the right foot on the second. The right column (**D**) shows the other valid pattern.

For both tasks, the time window of the analysis was a gait cycle defined by two consecutive foot contacts on the same side. Within the gait cycle, two time points were of interest. The time point on the way to the step (see Figure 2B(S1)) and the time point on the elevated surface (Figure 2B(S2)) (and their equivalents when walking on level ground (see Figure 2A(S1,S2)). A successful measurement was defined by the recording of the ground reaction forces with correct placement of the feet. One foot at a time had to be in complete contact with the force plate or step. For the two valid foot positions, it was important to check that no double contact (two touches of a force plate) occurred (see Figure 2C,D). In order to not influence the gait cycles of the subjects, they were not told the criteria of a successful measurement. Invalid measurements were sorted out afterwards. This procedure was necessary because the force of the foot–ground interaction was only known for the period in which the subject had exclusive contact with the force plate. Each individual trial thus covered the period from toe off of the contralateral foot to heel contact of the reference foot (foot on the first force plate). The same criteria applied to crossing of the step. Before starting the recordings, the test subjects had to complete a few test runs. On the one hand, this allowed them to get used to walking with the markers as well as to the test the track and crossing the step. On the other hand, a suitable starting point was determined so that foot contact took place completely on the measuring surface of the force plate in each case. Measurements were then taken until 20 valid trials could be recorded with an equal split between the two patterns of foot placement. Thus, at least 10 right-left-right and 10 left-right-left double-step sequences were recorded for each experimental condition. In the later simulation, both gait patterns were combined and thus the motion data for 10 complete gait cycles were presented.

### 2.8. Measurement Systems and Data Processing

The following section explains the technical details of the measurements performed during the experiment to obtain the motion data and ground reaction forces. In addition, the further processing of these data is explained in more detail.

1. Motion measurement (motion capture)

The subjects’ kinematic data were measured using a three-dimensional optical motion capture system comprising 8 infrared cameras (Oqus, Qualisys AB, Gothenburg, Sweden), 53 retroreflective markers (Qualisys AB, Ø 19 mm, Gothenburg, Sweden), and compatible data acquisition software (Qualisys Track Manager, Qualisys AB, Gothenburg, Sweden). Using the cameras, the markers were illuminated and their reflectance was recorded and transmitted to the computer. By combining the individual camera images, the 3D position of the markers could be calculated and a figure representing the subject’s movements in space could be reconstructed. In addition, a reference video camera (Sony, HDR-CX240E, 50 Hz) was connected to the measurement system, which recorded the trials and provided a means for subsequent visual inspection of individual shots. This made it possible to determine when participants’ feet made contact with the force plates. This information was critical for subsequent inverse dynamics analysis. Kinematic data were recorded at 100 Hz.

2. Measurement of Ground Reaction Force

Ground reaction forces were measured using two 600 × 400 mm force plates (4060-07, Bertec Corporation, Columbus, OH, USA) and compatible amplifiers (AM6501, Bertec Corporation, Columbus, OH, USA). The force plates were integrated into the test section at floor level. Two narrow floor elements could be removed from the side of the second force plate. This allowed the step to be mounted accurately on the force plate and fixed laterally by screws. Recording of the ground reaction forces was synchronized with the corresponding recording of the kinematic data since both were measured with the same acquisition software. The ground reaction forces were recorded at 1000 Hz.

3. Data processing

In this step, the measured motion data and ground reaction forces were prepared for use by the OpenSim simulation program. Qualisys computer software (Qualisys Track Manager, Qualisys AB, Gothenburg, Sweden) was used for further processing of the measured motion data. The reconstructed marker positions were named in the data acquisition software using the marker log. Gaps in the trajectories of the markers were filled by interpolation using the gap-filling function of Qualisys. Ground reaction forces were also acquired by Qualisys and linked to the motion data. A toolbox (MOtoNMS [matlab MOtion data elaboration TOolbox for NeuroMusculoSkeletal applications], version 2.2) freely available for the commercially available computer program Open Matlab (Release 2015b, TheMathWorks Inc., Natick, MA, USA) was used to process these data. This tool processed the experimentally recorded data and generated input data for optimal integration into a neuromusculoskeletal simulation program such as OpenSim (Mantoan et al. 2015). Data were filtered using an integrated low-pass filter (6 Hz) to minimize noise.

### 2.9. Simulation with OpenSim

Open Sim (version 3.3) is freely available software for modeling, simulation, and analysis of the neuromusculoskeletal system. Dedicated tools are available to create patient-specific simulations that allow meaningful information to be extracted from acquired motion data and ground reaction forces. These tools include scaling of an existing model, inverse kinematics, and inverse dynamics [13].

1. The model

The generic musculoskeletal model used was the “Gait Model 2392” developed by Darryl Thelen, Ajay Seth, Frank C. Anderson, and Scott L. Delp and freely available in OpenSim. This three-dimensional model can be used and modified to calculate the joint angles and moments of a subject during movement and to study the influence of surgical interventions on the musculoskeletal system [13,14].

The generic model was primarily a lower extremity model, with two legs and a clenched trunk segment that included the weight of the arms. It was composed of 12 rigid body segments, consisting of the torso (spine, thorax, cranium), pelvis, bilateral femur, tibia, calcaneus, talus, and digiti pedis, respectively, with a height of 1.80 m and weight of 75.16 kg. The segments were connected by joints. There were a total of 23 degrees of freedom. The hip joint was modeled as a spherical joint with 3 degrees of freedom, the axes of which were located in the femoral head. This allowed rotation, adduction/abduction, and flexion/extension. For the knee joint, a simplified model with only one degree of freedom was used, as developed by Yamaguchi and Zajac [15]. In this model, the femoral condyles were represented as ellipses and the tibial plateau was planar. The femoral condyles were in contact with the tibial plateau throughout the time of knee motion. The tibiofemoral contact point depended on the knee angle. The model allowed flexion and extension. The ankle joints were modeled as frictionless rotational joints.

Six of the existing 23 lines of freedom represented the lines of freedom (three translational axes and three rotational axes) between the model’s pelvis and the ground, referred to as residual joints. The model was connected to the environment via the inertial system of the pelvis, which was located midway between the spinae iliacae anteriores superiores. The forces of the residual joints that activated translation between the pelvis and ground were called residual forces, and the forces that activated rotation were called residual moments.

The generic model had a set of virtual markers placed at the same anatomical locations as the experimental markers to allow the recorded motions to be transferred to the model.

2. Scaling

The specific modeling involved scaling of the generic model and registration of the markers placed on the model. Scaling was performed to transfer the anthropometric data of the subject to the generic model. This was essential to obtain the most accurate analysis data possible from the transferred movements. The scaling step adapted the mass properties of the generic model and dimensions of the body segments. The first step was to calculate the scaling factors for each body segment. Each segment was defined by a selected pairs of markers. The data of the experimental markers came from the static measurements. The distance between a pair of markers was considered for each frame of an image to calculate an average value. In measurement-based scaling, the ratio between the distance of an experimental marker pair and the distance of the corresponding virtual marker pair gives a scaling factor. To improve the accuracy of scaling, a segment can also be defined by multiple marker pairs from which the scaling factor is calculated. The total scaling factor is then the average of the scaling factors calculated based on all pairs. Based on the calculated factors, the geometry of the model was scaled. The process was performed for each body segment in the three spatial axes.

The next step was to adjust the position of the virtual markers to better match the position of the experimental markers. To do this, the model was placed in a position that corresponded to the position of the subject during the static experiment. Once a static pose was computed, all model markers were moved to the position of the experimental markers. The smallest possible distance between the virtual and experimental markers obtains the best possible results (specification in the OpenSim documentation: maximum difference < 2 cm, Root Mean Square (RMS) < 1 cm). For this purpose, a relative weight was assigned to each marker. Marker weights determined how well the virtual markers tracked the trajectories of the experimental markers. A larger weight called for a more accurate match between the corresponding virtual and experimental marker positions. The resulting scaled model was used for further simulation steps and analysis.

3. Inverse Kinematics (IK)

In this analysis step, using the scaled model and the experimentally collected gait data, the joint angles of the subjects during movement were calculated. The experimental markers were matched with the virtual markers throughout the movement by varying the joint angles over time. Inverse kinematics cycled through each time point of the motion and placed the model in a pose that best matched the experimental marker positions for that time point. Mathematically, the “best fit” was expressed as a weighted least squares function (Equation (1)), the solution of which aimed to minimize marker error. Marker error was defined as the distance between an experimental marker $x_{i}^{exp}$ and the corresponding virtual marker $x_{i}$. Each marker was assigned a weight, $w_{i}$, which indicated how much the error of the marker should be minimized by the least squares function. Relevant markers were assigned higher weights. The resulting vector, q, contained the joint angle for each degree of freedom.

(1) $$\underset{q}{\min}\left[\underset{i\epsilon Marker}{\sum }w_{i}{||x_{i}^{exp}-x_{i}\left(q\right)||}^{2}\right]$$

To evaluate the results, the marker errors recommended in the OpenSim document (maximum error < 4 cm, RMS < 2 cm) were considered and the joint angle curves obtained were compared with reference curves from the literature [16,17].

4. Inverse dynamics (ID)

Inverse dynamics was used to calculate the net reaction forces and moments that occurred during the recorded motion for each joint. To determine these forces, stepwise equations of motion were solved using the joint positions, velocities, and accelerations known from inverse kinematics, the experimental ground reaction forces, and the scaled model. The following equation (Equation (2)) underlies the inverse dynamics algorithm:

(2) $$\vec{\tau }=M\left(\vec{q}\right)⬝\ddot{\vec{q}}+\vec{C}\left(\vec{q},\dot{\vec{q}}\;\right)+\vec{G}\left(\vec{q}\right)$$

The variables $\vec{q}$, $\ddot{\vec{q}}$, and $\dot{\vec{q}}$ represent the location, velocity, and acceleration of the joints, respectively; $M\left(\vec{q}\right)$ represents the mass properties of the model; $\vec{C}\left(\vec{q},\dot{\vec{q}}\;\right)$ represents the vector of Coriolis and centrifugal forces; $\vec{G}\left(\vec{q}\right)$ represents the vector of gravitational forces; and $\vec{\tau }$ represents the vector of generalized joint forces.

The motion of the model was completely determined by the variables $\vec{q}$, $\ddot{\vec{q}}$, and $\dot{\vec{q}}$ (joint angle, velocity, acceleration, respectively). Consequently, these variables were known and the inverse dynamics could be used to solve the equation for the unknown variable, $\vec{\tau }$.

The calculated torques of the joints were compared with data from the literature [16,17] to check the simulation quality. The joint force curves were low-pass filtered at 6 Hz to minimize artifacts.

5. Residual Reduction Algorithm (RRA)

In the following, the application of the residual reduction algorithm (RRA) is explained. The RRA is a forward dynamic simulation that can be applied to measured motions in which a model is displaced relative to the ground while subjected to ground reaction forces and torques. Each of the 23 lines of freedom of the model was assigned an actuator; a distinction was made between joint actuators and actuators for the six residual forces, F_x, F_y, F_z, M_x, M_y, and M_z.

The goal of using RRA is to minimize the effects of inaccuracies in the model geometry (model without arms) or mass distribution, noise, or errors from motion capture data. These can accumulate and result in large non-physical forces called residuals that can lead to dynamic inconsistency between the measured ground reaction forces and moments and the model kinematics. Due to these inconsistencies, Newton’s 2nd law (F = m⬝a) cannot be satisfied.

By adding the six residual forces to the equation, this inconsistency was accounted for and the equation was adjusted as follows (see Equation (3)):

(3) $$F+F_{Resudial}=m⬝a$$

The residual forces should be reduced to the absolute minimum to prevent them from producing forces that should actually be assumed by the joint actuators. In order to obtain the most realistic forces of the joint actuators, this possible source of error is minimized by applying the RRA and the resulting adjustment of the center of mass and kinematics, as well as keeping the limits.

At the beginning of the simulation, the model was placed in the initial position calculated by the inverse kinematics for the start time. The RRA then calculated stepwise (0.001 s) the forces of all actuators necessary to move the model to the final position. The following objective function (Equation (4)) was minimized for the calculation:

(4) $$J=\overset{nx}{\underset{i=1}{\sum }}x_{i}{}^{2}+\overset{nq}{\underset{j=1}{\sum }}w_{j}⬝{\left({\ddot{q}}_{j}{}^{*}-{\ddot{q}}_{j}\right)}^{2}$$

In the objective function (J), the sum of squared actuator activation values, $x_{i}$, was added to the sum of the squared and weighted acceleration errors formed by the difference of the target acceleration of the respective line of freedom, ${\ddot{q}}_{j}{}^{*}$, and the current acceleration of the same line of freedom, ${\ddot{q}}_{j}$. The factor $w_{j}$ determined the weighting of the freedom line.

At the end of the simulation, the mean value of the actuators of the residual forces was calculated. The mean values of the residual forces, M_x_ (left–right rotation) and M_z_ (forward–backward rotation), were used to adjust the center of mass to correct for excessive “skewness” of the model due to inaccuracies in the mass distribution and geometry of the torso in the model.

The result of the RRA is an adjustment to the kinematics and center of mass, which reduces the residual forces that do not occur during motion. However, this may result in a slightly different motion compared to that calculated by the inverse kinematics.

### 2.10. Statistics

**Figure 3:**
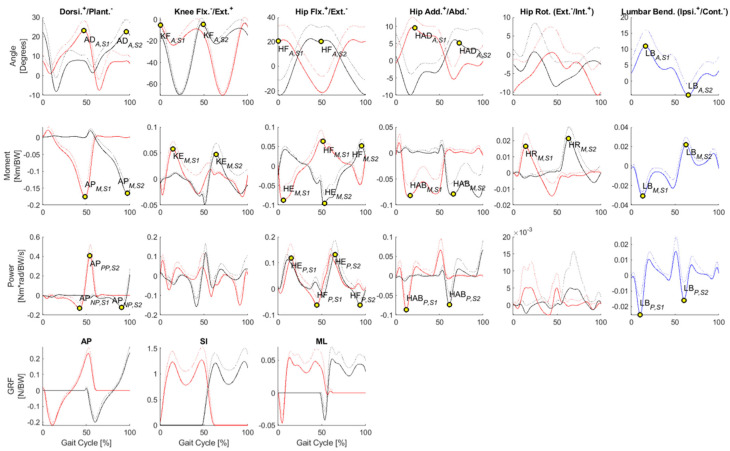
Selected joint parameters. The values of parameters are indicated by circular markers on the group means of joint angles, moments, and power during level-ground walking by the control group. Solid and dotted lines respectively indicate inter-participant mean and standard deviation. Red lines indicate the leading limb at the onset of S1, while black lines indicate the lagging limb. Blue lines indicate lumbar bending. The y-axis shows the respective joint angles, joint moments, joint power, and ground reaction forces, while the x-axis shows the gait cycle [%]. A = joint angle, AD = ankle dorsiflexion, AP = ankle plantarflexor (in diagram with moment and power on the y-axis) or anteroposterior (in diagram with GFR on the y-axis), GRF = ground reaction force, HAB = hip abductor moment, HAD = hip adduction, HE = hip extensor, HF = hip flexion, HR = hip external rotator, KF = knee flexion, KE = knee extensor, LB = lumbar bending, M = joint moment, ML = medio lateral, NP = negative joint power, P = joint power, S1 = during S1, S2 = during S2, SI = superior inferior.

**Figure 4:**
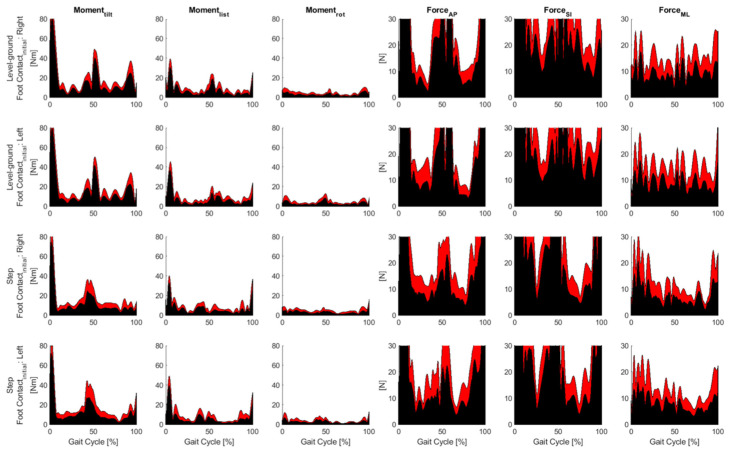
Residual moments and forces at the pelvis during inverse dynamics for the control group. The tilt moment is calculated about the mediolateral (ML) axis, the list moment is calculated about the anteroposterior (AP) axis, and the rotational (rot) moment is calculated about the superior inferior (SI) axis. Red represents the standard deviation, black represents the mean values.

Matlab was used for statistical analysis of the data. The parameters analyzed included gait speed and stride length, as well as lower extremity joint angles, moments, and forces, and mediolateral lumbar flexion. Joint parameters were selected based on their inter-trial and inter-participant waveform consistency and previous reports of task-related discrepancies or effects of iliosacral fixation. The selected joint parameters are listed below and also indicated on the waveforms of joint angles, moments, and power in Figure 3 and Figure 4.

Each of these parameters was analyzed using a mixed analysis of variance (ANOVA) with the trial condition as the within-subject factor (trial task and standing leg) and the group as the between-subject factor. The significance level was set at *p* ≤ 0.05 for all tests.

– Joint angles
  ○ Peak mediolateral lumbar bending during S1 (LB*_A_*_,*S*1_)
  ○ Peak mediolateral lumbar bending during S2 (LB*_A_*_,*S*2_)
  ○ Hip flexion at the start of S1 (HF*_A_*_,*S*1_)
  ○ Hip flexion at the start of S2 (HF*_A_*_,*S*2_)
  ○ Peak hip adduction during S1 (HAD*_A_*_,*S*1_)
  ○ Peak hip adduction during S2 (HAD*_A_*_,*S*2_)
  ○ Knee flexion at the start of S1 (KF*_A_*_,*S*1_)
  ○ Knee flexion at the start of S1 (KF*_A_*_,*S*2_)
  ○ Peak ankle dorsiflexion during S1 (AD*_A_*_,*S*1_)
  ○ Peak ankle dorsiflexion during S2 (AD*_A_*_,*S*2_)
– Joint moments
  ○ Peak mediolateral lumbar bending moment during S1 (LB*_M_*_,*S*1_)
  ○ Peak mediolateral lumbar bending moment during S2 (LB*_M_*_,*S*2_)
  ○ Peak hip extensor moment during S1 (HE*_M_*_,*S*1_)
  ○ Peak hip extensor moment during S2 (HE*_M_*_,*S*2_)
  ○ Peak hip flexor moment during S1 (HF*_M_*_,*S*1_)
  ○ Peak hip flexor moment during S2 (HF*_M_*_,*S*2_)
  ○ First peak hip abductor moment during S1 (HAB*_M_*_,*S*1_)
  ○ First peak hip abductor moment during S2 (HAB*_M_*_,*S*2_)
  ○ Peak hip external rotator moment during S1 (HR*_M_*_,*S*1_)
  ○ Peak hip external rotator moment during S2 (HR*_M_*_,*S*2_)
  ○ Peak knee extensor moment during S1 (KE*_M_*_,*S*1_)
  ○ Peak knee extensor moment during S2 (KE*_M_*_,*S*2_)
  ○ Peak ankle plantarflexor moment during S1 (AP*_M_*_,*S*1_)
  ○ Peak ankle plantarflexor moment during S2 (AP*_M_*_,*S*2_)
– Joint power
  ○ Peak negative mediolateral lumbar bending power during S1 (LB*_P_*_,*S*1_)
  ○ Peak negative mediolateral lumbar bending power during S2 (LB*_P_*_,*S*2_)
  ○ Peak positive hip extensor power during S1 (HE*_P_*_,*S*1_)
  ○ Peak positive hip extensor power during S2 (HE*_P_*_,*S*2_)
  ○ Peak negative hip flexor power during S1 (HF*_P_*_,*S*1_)
  ○ Peak negative hip flexor power during S2 (HF*_P_*_,*S*2_)
  ○ Peak negative hip abductor power during S1 (HAB*_P_*_,*S*1_)
  ○ Peak negative hip abductor power during S2 (HAB*_P_*_,*S*2_)
  ○ Peak negative ankle plantarflexor power during S1 (AP*_NP_*_,*S*1_)
  ○ Peak negative ankle plantarflexor power during S2 (AP*_NP_*_,*S*2_)
  ○ Peak positive ankle plantarflexor power during S1 (AP*_PP_*_,*S*1_)

## 3. Results

### Analyzed Joint Parameters

The results in Figure 3 show selected joint parameters during ground-level walking in the control group. The data indicated that there was no difference between the right and left extremities in the healthy gait pattern and the curves for the corresponding joint angles and moments. The joint forces showed the same progression within a gait cycle but were offset. The red lines mark the anterior limb at the beginning of the gait cycle at S1, while the black lines show the posterior limb at the same time. The blue lines indicate the lumbar flexion of the spine within this gait cycle. The ground reaction force (GRF) indicates the force that the ground exerts on a body that comes into contact with it.

During inverse kinematics, we calculated the difference between the experimental and model marker trajectories for each marker over a boney landmark. The inter-participant mean maximum difference in the two groups for the two experimental tasks was below 25 mm for all markers over boney landmarks except for the markers over the medial and lateral epicondyles of the femur, indicating that inter-participant mean maximum discrepancies were below 40 mm. The corresponding inter-participant mean of the root mean square of the difference was 20 mm or below 20 mm for both the patients and healthy study participants during the two experimental tasks.

Table 2 represents an overview of the significant differences between the stance limb and gait leg measurements. It shows that all parameters except HE*_P_*_,*S*1_ (not listed) were significantly different. The data showed that the measurements of the forces in the area of lumbar bending were significantly lower for level-ground walking than for step crossing. The only exception was the measurement of joint angles, where a higher value was found in favor of level-ground walking. As expected, a similar difference was found for hip flexion and extension. Specifically, within the gait cycle, the patients exhibited reduced hip adduction during step (*p* = 0.058), increased hip abduction during swing (p ** = 0.002), reduced foot dorsiflexion during stance (p ** = 0.003), reduced hip extensor moment during early stance (p *** = 0.001), as well as increased hip flexor moment during late stance to early swing (p ** = 0.006). The other individual parameters are not listed any further and can be taken from Table 2. Table 3 shows the normalized walking speed and step length (measured from the onset of S1 to the onset of S2) of the two groups. The walking speed was slower when walking over an elevated surface compared to level-ground walking (*F*_1,14_ = 56.9, p ^***^ < 0.001, 95% CI: 7.75–13.91%*_Height_*). The speed was not affected by the leading limb (*F*_1,14_ = 0.017, *p* = 0.897) or the ISF (*F*_1,14_ = 0.834, *p* = 0.377). Also, the step length was not significantly affected by the ISF (*F*_1,14_ = 2.51, *p* = 0.135), the experimental task (*F*_1,14_ = 4.42, *p* = 0.054), or the leading limb (*F*_1,14_ = 0.167, *p* = 0.689).

The speed and step length were normalized to the height of each participant. In each cell, the values are shown as inter-participant mean ± standard deviation. The speed and step length are shown separately for trials with either leading limb: right or left for the control group and fixed or free for the iliosacral fixation (ISF) group. Step length was measured between the onsets of S1 and S2.

**Figure 5:**
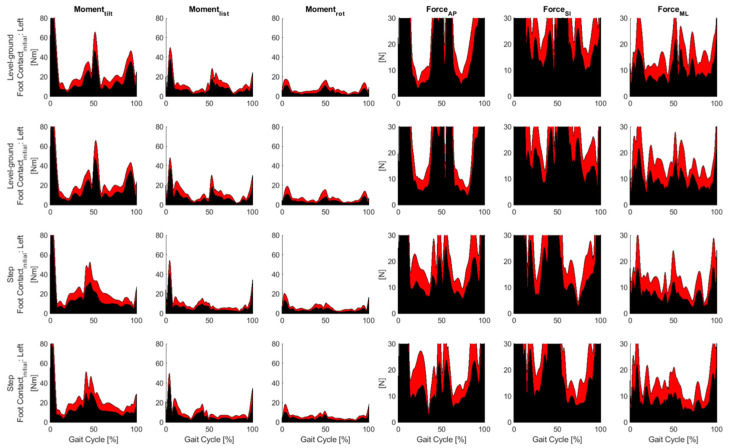
Residual moments and forces at the pelvis during inverse dynamics for the ISF group. The tilt moment is calculated about the mediolateral (ML) axis, the list moment is calculated about the anteroposterior (AP) axis, and the rotational (rot) moment is calculated about the superior inferior (SI) axis. Red represents the standard deviation, black represents the mean values.

Figure 4 and Figure 5 show the residual moments and forces at the pelvis during inverse dynamics for the control and ISF groups, respectively. In inverse dynamics, the forces underlying the motion are derived. From information about the velocity in combination with assumptions about the mass of the body parts, the acting forces at each point of the body at each point in time were estimated. The so-called residual forces indicated the corresponding deviations, i.e., how far the calculations were from a perfect representation of the measurement. The graphs in red represent the standard deviation, while those in black indicate the mean values. The graphs show for which phase of movement (from 0–100%) the calculation was accurate (small residual forces), and where forces were not correctly represented by the model and how large they were. OpenSim recommends a maximum residual moment of less than 75 Nm (ideally less than 50 Nm) and a maximum residual force of less than 25 N (ideally less than 10 N). For level ground and step, no difference could be seen in the range of different torques and forces acting on the right and left legs during foot contact in the control group (left side of Figure 4). On the right side of Figure 4, the forces [N] are shown for one gait cycle each for step and level ground divided into three axes.

The torque decreased continuously for both extremities from tilt moment via list moment to rotation (rot) moment. In the distribution of forces between extremities, there was no difference between the right and left extremities for the individual force directions indicated.

As in Figure 4, Figure 5 shows the respective forces and torques for the ISF group. However, it can be seen that the residual forces after ISF were higher than in the control group. This meant that the deviations of the calculations after iliosacral joint fixation were further away from a “perfect” representation of the measurement as in the control group, as expected.

**Figure 6:**
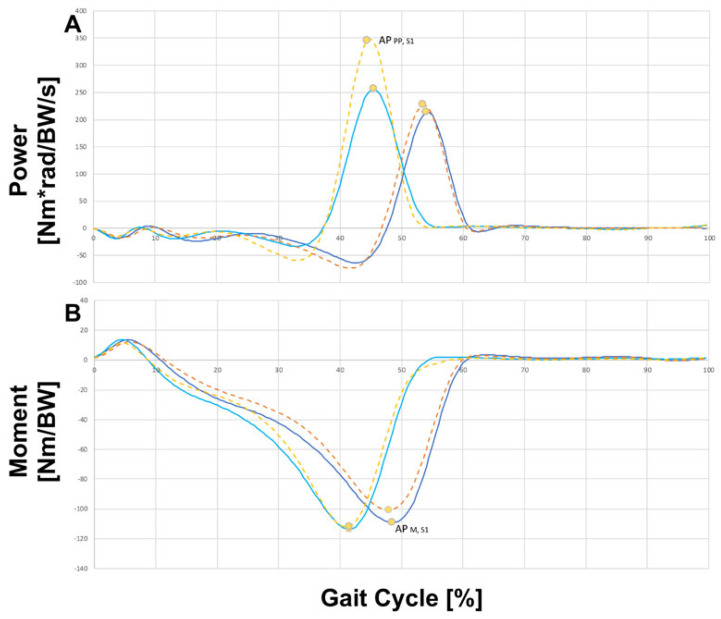
Selected joint parameters. The values of parameters are indicated by circular markers on the group means of joint angles, moments, and power during level-ground walking and stepping onto the elevated surface by the control group and iliosacral fixated (ISF) joint group in comparison. (**A**) Power of the gait and step in the control group vs. ISF group related to the plantar and dorsal flexors. Yellow dashed line shows the step for the control group, orange dashed line shows the walk for the control group, light blue line shows the step for the ISF group, dark blue line shows the walk for the ISF group. The y-axis shows the power [Nm*rad/BW/s], while the x-axis shows the gait cycle [%]. (**B**) Moments of the gait and step in the control group vs. ISF group related to the plantar and dorsal flexors. Yellow dashed line shows the step for the control group, orange dashed line shows the walk for the control group, light blue line shows the step for the ISF group, dark blue line shows the walk for the ISF group. The y-axis shows the moment [Nm/BW], while the x-axis shows the gait cycle [%]. AP = ankle plantarflexor, M = joint moment, P = joint power, S1 = during S1.

The results also showed that all selected joint parameters, except the “peak negative hip flexor power during S1” (HE*_P_*_,*S*1_), showed a difference between patients and control individuals (Figure 3 and Figure 6). This finding was consistent with a previous study comparing walking on level ground and stair climbing in nondisabled participants [1]. The force of the plantar flexors of the control individuals, i.e., those without iliosacral joint fixation, was greater during push-off while stepping on the elevated surface (see Table 2). Extended forward thrust appeared to be needed to bring the body onto the elevated surface with the contralateral foot. In addition, during stance on the elevated surface (S2), hip flexion and the corresponding hip extensor moment, knee flexion and the corresponding knee extensor moment, and ankle dorsiflexion as well as the corresponding negative plantar flexor force were increased (see Table 2). Thus, the limbs of patients needed to be flexed more on the elevated surface to prevent collapse than was the case on level ground.

The main difference between the control group and the iliosacral fixated (ISF) joint group was in the application of the power of the ankle (see Figure 6); we can see this in Figure 6A where the yellow dashed line of the step for the control group shows that significantly more force was applied than in the ISF group. This meant that patients after ISG fixation were significantly less able to transfer power into mobilization onto the step than the control group without ISG fixation. This was indeed the major difference observed in mobilization, whereas no significant difference was found with respect to force/moment measurements between the group of patients and the healthy group of people. In the healthy control group, a value of approx. 350 N was found, while this value was significantly lower in the ISG fixation group with approx. 260 N (p ** = 0.002). These results showed that the elastic effect of the iliosacral joint appeared to be lost due to screw fixation.

## 4. Discussion

During the gait cycle, as defined as the time period between successive heel contacts of the same foot [8], the iliosacral joint allows relative rotation between the hip bones and sacrum. During this process, the hip bone rotates posteriorly in the sagittal plane relative to the sacrum on the side of the swinging leg, and it rotates anteriorly in the sagittal plane relative to the sacrum on the side of the stance leg [8]. These rotations have been noted during hip flexion and extension in the supine position, which are analogous to the swing and stance phases of the gait cycle, respectively [9]. Our study indicates that elimination of these rotations by immobilizing the iliosacral joint alters the gait pattern. It is well known that there are limited mobility and movement in the iliosacral joint during well controlled leg movements. However, it has been questioned whether these limitations apply during walking, i.e., during which muscle activity is much higher and could further stiffen the iliosacral joint. Our results suggest that this is indeed the case after iliosacral screw fixation.

The effects of iliosacral joint immobilization can be inferred from gait impairments after internal fixation of an unstable pelvic ring disruption [1]. Some previous studies have reported that individuals with internally fixed pelvic ring disruptions have a shorter mean stride length with greater inter-individual variability when compared to normative data [3,4], whereas other studies have reported the mere presence of gait impairments [10] or simple descriptions based on visual inspection [11]. A first more detailed study by Kubota et al. [18] focused on participants who had undergone open reduction and internal fixation of pelvic fractures followed by a conventional rehabilitation program [18]. The authors noted that twelve months after surgery, the patients exhibited normal walking speed and stride length and asymmetry in the strength of the hip flexors, adductors, and abductors was significantly reduced [18]. Despite this apparent normalization, the patients exhibited kinematic and kinetic abnormalities compared to controls [18]. Specifically, within the gait cycle, the patients exhibited reduced hip adduction during stance (*p* = 0.058), increased hip abduction during swing (*p* = 0.002), reduced foot dorsiflexion during stance (p ** = 0.003), reduced hip extensor moment during early stance (p *** = 0.001), as well as increased hip flexor moment during late stance to early swing (p ** = 0.006) [18]. The magnitude of the observed abnormalities was comparable to those in other pathologies, such as chronic rheumatoid arthritis [19], stroke [20], or Parkinson’s disease [21,22]. Although 4 of the 18 participants were treated for stable and minimally displaced pelvic ring fractures, the above observations already indicate the effects of iliosacral immobilization on gait [12]. They suggest immobilization of the sacroiliac joint may also affect pelvic and lower limb movements during gait. These movements allow efficient bipedal walking by generating a low-amplitude sinusoidal trajectory of the human body’s center of gravity (COG) [23]. They are often referred to as the six main determinants of gait [23]: (i) pelvic rotation in the transverse plane relative to the direction of progression; (ii) pelvic tilt in the coronal plane relative to the horizontal, which is accompanied by hip adduction in stance and hip abduction in swing; (iii) knee flexion during stance; (iv) foot rotations during stance; (v) knee flexion associated with foot rotations during stance; and (vi) lateral displacement of the pelvis (reduced by the tibiofemoral angle and relative hip adduction during stance). The first three of these determinants (i–iii) reduce the vertical displacement of the COG, (iv) and (v) smooth the trajectory of the COG in the sagittal plane, and the last determinant (vi) governs the mediolateral trajectory of the COG [23]. The findings of Kubota et al. [18] suggest that iliosacral joint immobilization affects the determinants (ii), (iv), and (vi). This conclusion is consistent with the general assumption that immobilization of the iliosacral joint can alter the kinematic and kinetic parameters of gait. However, to clearly describe the effects of joint immobilization, it is necessary to investigate whether such abnormalities still persist after the joint immobilization has been released. Therefore, we focused on healthy participants in our study and need to follow-up with patients in further studies just before and after removal of joint-immobilizing implants.

The results highlight that significant group differences were only found in the peak plantarflexor moment and corresponding push-off power during S1, with the ISF group generating less moment and power during both experimental tasks (Table 1). These differences were not observed during level-ground walking but were pronounced when the participants were stepping onto the elevated surface. The average group differences were 0.007 Nm/BW and 0.036 Nm⋅rad/BW/s for level-ground walking and 0.016 Nm/BW and 0.148 Nm⋅rad/BW/s for stepping onto the elevated surface, respectively. These differences suggest that individuals with iliosacral fixation have reduced forward propulsion during ambulation particularly on a terrain requiring greater range of hip motion in the sagittal plane and that the elastic effect of the iliosacral joint has been lost due to screw fixation. This finding is even more intriguing as there is no anatomically intuitive link between fixation of the iliosacral joint and actuation of the ankle. The results therefore encourage further studies to investigate whether the reduced propulsion reflects a physiological impairment and whether a post-operative strategy requiring participants to perform more challenging tasks, such as moving as fast as possible rather than letting them walk at their self-selected speed, should be adopted. The clinical significance of the impairment has not yet been studied in detail. However, it was observed that the participants in the ISF group walked slower on average than those in the control group (Table 2), even though this result was not statistically significant.

A previous study addressed only level-ground walking between a post-operative pelvic ring fracture group and control group [2]. The authors reported reduced stance ankle dorsiflexion, reduced early-stance hip extensor moment, as well as increased late-stance hip flexor moment for the ISF group [2]. The results of our study confirm these previous findings and extend them to include asymmetry in the negative mediolateral lumbar bending power during S1 and S2, positive hip extensor power during S2, negative hip abductor power during S1, and negative plantarflexor power during S1 and S2. There was no inherent characteristic of the control group that could be used to dichotomously categorize their limbs. Thus, it is difficult to interpret the observed asymmetry. Interestingly, the negative hip abductor power and negative plantarflexor power of individuals in the ISF group were extended on the free side while the positive hip extensor power was greater on the fixed side. Surprisingly, the asymmetry of the lumbar bending power was contradictory, as the side with greater power was different between S1 and S2. However, asymmetry as a fixation-specific effect on gait cannot be excluded, although it is difficult to infer the nature of these effects from the present results.

Our study has some limitations, including the relatively low number of patients. It should be noted, however, that the number of patients with unilateral screw fixation of the posterior pelvic ring is generally low. Also, we did not perform a power analysis in our study, which is usually performed before data collection. This provides an estimate of how many individuals are needed to perform the study or once the actual data are collected and no significant difference is observed. The injuries that were examined in our study are rare and we faced only a small patient population. However, the data provided by the small patient population already showed a statistically significant difference; thus, a power analysis after data collection was not necessary.

## 5. Conclusions

Our study provides extended insight into the impact of current surgical treatment of unstable pelvic ring injuries on patient gait. As gait has been shown to be impaired following iliosacral screw fixation, this may not only impact patient independence and quality of life, but any abnormal movement may lead to secondary musculoskeletal complications, resulting in pain. This is particularly relevant for individuals who are not candidates for implant removal after fracture healing owing to their advanced age. As shown here, gait abnormalities can be monitored, analyzed for specific and objective parameters, and then targeted for treatment. Further studies with larger patient populations are needed to further investigate and elucidate the role of the iliosacral joint in bipedal walking in detail. The question of whether patients with or without iliosacral screw fixation show different results should become the target of further studies including additional measurement approaches of gait analysis. In fact, we have already used a novel analysis system (X-Sense suit with MVN motion system) to objectively compare the gait patterns of patients and healthy individuals. Such an analysis will provide deeper and objective insight into the individual movement patterns that also include muscle and joint loads during the walking process.

## Figures and Tables

**Table 1:** Baseline characteristics of the populations.

Description	ISF Group (*n* = 8)	Control Group (*n* = 8)
Number of patients	8	8
Age range [years]	18–68	19–69
Age mean [years] ± SD	45.63 ± 23.19	46.50 ± 22.91
Body height mean [cm] ± SD	176.50 ± 12.99	170.50 ± 10.70
Body weight mean [cm] ± SD	71.88 ± 12.02	64.75 ± 12.83
BMI mean [kg/m^2^] ± SD	23.03 ± 2.45	22.51 ± 5.77
**Gender**		
Female [*n*]	5	5
Male [*n*]	3	3

ISF = iliosacral fixation; BMI = Body Mass Index.

**Table 2:** Significant main effects of the experimental conditions (task and stance limb) and unilateral ISF on the joint parameters.

Parameter	Main Effect	Comparison	95% CI
LB*_A_*_,*S*1_	Task: *F*_1,14_ = 34.2, p *** < 0.001	LG > STEP	1.02–2.20 degrees
LB*_A_*_,*S*2_	Task: *F*_1,14_ = 54.0, p *** < 0.001	LG < STEP	2.98–5.46 degrees
LB*_M_*_,*S*1_	Task: *F*_1,14_ = 23.5, p *** < 0.001	LG < STEP	0.004–0.010 Nm/BW
LB*_M_*_,*S*2_	Task: *F*_1,14_ = 12.9, p ** = 0.003 Stance limb: *F*_1,14_ = 5.4, p * = 0.037	LG < STEP Right < Left; Fixed > Free	0.003–0.010 Nm/BW 4.30 × 10^−4^–0.012 Nm/BW
LB*_P_*_,*S*1_	Stance limb: *F*_1,14_ = 5.0, p * = 0.043	Right > Left; Fixed < Free	2.26 × 10^−4^–0.013 Nm⋅rad/BW/s
LB*_P_*_,*S*2_	Task: *F*_1,14_ = 22.5, p *** < 0.001 Stance limb: *F*_1,14_ = 5.8, p * = 0.032	LG > STEP Right < Left; Fixed > Free	0.006–0.015 Nm⋅rad/BW/s 4.93 × 10^−4^–0.009 Nm⋅rad/BW/s
HF*_A_*_,*S*1_	Task: *F*_1,14_ = 10.5, p ** = 0.007	LG < STEP	0.45–2.27 degrees
HF*_A_*_,*S*2_	Task: *F*_1,14_ = 822.3, p *** < 0.001 Stance limb: *F*_1,14_ = 6.4, p * = 0.025	LG < STEP Right < Left; Fixed > Free	32.40–37.68 degrees 0.11–1.43 degrees
HE*_M_*_,*S*1_	Task: *F*_1,14_ = 4.8, p * = 0.047	LG < STEP	8.15 × 10^−5^–0.011 Nm/BW
HE*_M_*_,*S*2_	Task: *F*_1,14_ = 114.0, p *** < 0.001	LG < STEP	0.040–0.060 Nm/BW
HF*_M_*_,*S*1_	Task: *F*_1,14_ = 115.8, p *** < 0.001	LG > STEP	0.029–0.044 Nm/BW
HF*_M_*_,*S*2_	Task: *F*_1,14_ = 124.7, p *** < 0.001	LG < STEP	0.041–0.060 Nm/BW
HE*_P_*_,*S*2_	Task: *F*_1,14_ = 144.9, p *** < 0.001 Stance limb: *F*_1,14_ = 6.9, p * = 0.021	LG < STEP Right < Left; Fixed > Free	0.141–0.203 Nm⋅rad/BW/s 0.001–0.132 Nm⋅rad/BW/s
HF*_P_*_,*S*1_	Task: *F*_1,14_ = 45.5, p *** < 0.001	LG > STEP	0.026–0.051 Nm⋅rad/BW/s
HF*_P_*_,*S*2_	Task: *F*_1,14_ = 72.0, p *** < 0.001	LG < STEP	0.053–0.089 Nm⋅rad/BW/s
HAD*_A_*_,*S*1_	Task: *F*_1,14_ = 66.1, p *** < 0.001	LG > STEP	1.86–3.21 degrees
HAD*_A_*_,*S*2_	Task: *F*_1,14_ = 11.3, p ** = 0.005	LG < STEP	0.52–2.40 degrees
HAB*_M_*_,*S*1_	Task: *F*_1,14_ = 25.4, p *** < 0.001	LG < STEP	0.005–0.011 Nm/BW
HAB*_M_*_,*S*2_	Task: *F*_1,14_ = 196.1, p *** < 0.001	LG > STEP	0.021–0.029 Nm/BW
HAB*_P_*_,*S*1_	Stance limb: *F*_1,14_ = 7.3, p * = 0.018	Right > Left; Fixed < Free	0.002–0.017 Nm⋅rad/BW/s
HAB*_P_*_,*S*2_	Task: *F*_1,14_ = 101.4, p *** < 0.001	LG > STEP	0.038–0.059 Nm⋅rad/BW/s
HR*_M_*_,*S*1_	Task: *F*_1,14_ = 7.5, p * = 0.017	LG < STEP	3.43 × 10^−4^–0.003 Nm/BW
HR*_M_*_,*S*2_	Task: *F*_1,14_ = 55.6, p *** < 0.001	LG < STEP	0.005–0.009 Nm/BW
KF*_A_*_,*S*1_	Task: *F*_1,14_ = 31.2, p *** < 0.001	LG < STEP	1.21–2.74 degrees
KF*_A_*_,*S*2_	Task: *F*_1,14_ = 1560.3, p *** < 0.001	LG < STEP	42.56–47.48 degrees
KE*_M_*_,*S*1_	Task: *F*_1,14_ = 13.5, p ** = 0.003	LG < STEP	0.004–0.017 Nm/BW
KE*_M_*_,*S*2_	Task: *F*_1,14_ = 34.8, p *** < 0.001	LG < STEP	0.020–0.041 Nm/BW
AD*_A_*_,*S*1_	Task: *F*_1,14_ = 20.2, p *** < 0.001	LG > STEP	1.73–4.93 degrees
AD*_A_*_,*S*2_	Task: *F*_1,14_ = 358.4, p *** < 0.001	LG < STEP	14.69–18.48 degrees
AP*_M_*_,*S*1_	Group: *F*_1,14_ = 4.7, p * = 0.049 Task: *F*_1,14_ = 50.8, p *** < 0.001	Control > ISF LG < STEP	2.97 × 10^−5^–0.023 Nm/BW 0.015–0.029 Nm/BW
AP*_M_*_,*S*2_	Task: *F*_1,14_ = 574.8, p *** < 0.001	LG > STEP	0.041–0.049 Nm/BW
AP*_NP_*_,*S*1_	Task: *F*_1,14_ = 10.6, p ** = 0.006 Stance limb: *F*_1,14_ = 6.7, p * = 0.022	LG > STEP Right > Left; Fixed < Free	0.009–0.042 Nm⋅rad/BW/s 0.002–0.018 Nm⋅rad/BW/s
AP*_NP_*_,*S*2_	Task: *F*_1,14_ = 8.9, p * = 0.011 Stance limb: *F*_1,14_ = 6.7, p * = 0.023	LG < STEP Right > Left; Fixed < Free	0.009–0.056 Nm⋅rad/BW/s 0.002–0.027 Nm⋅rad/BW/s
AP*_PP_*_,*S*1_	Group: *F*_1,14_ = 14.2, p ** = 0.002 Task: *F*_1,14_ = 61.7, p *** < 0.001	Control > ISF LG < STEP	0.039–0.144 Nm⋅rad/BW/s 0.161–0.283 Nm⋅rad/BW/s

A = joint angle, AD = ankle dorsiflexion, AP = ankle plantarflexor, BW = body weight, F = value for ANOVA, HAB = hip abduction, HAD = hip adduction, HE = hip extensor, HF = hip flexion, HR = hip external rotator, KF = knee flexion, LB = lumbar bending, LG = level-ground walking, M = joint moment, Nm = newton-meter, NP = negative joint power, P = joint power, rad = radiant, s = seconds, S1 = during S1, S2 = during S2, SI = superior inferior, STEP = stepping onto and down from an elevated surface. > = comparative value was greater, < = comparative value was smaller, significance levels: *** = p *** ≤ 0.001, ** = p ** ≤ 0.01, * = p * ≤ 0.05.

**Table 3:** Normalized walking speed and step length of the control and ISF groups.

Task	Group	Speed [%_height_/s]		Step Length [%_height_]	
		Right/Fixed	Left/Free	Right/Fixed	Left/Free
Level ground	Control	78 ± 6	78 ± 5	41 ± 2	41 ± 2
	ISF	75 ± 8	75 ± 10	40 ± 2	40 ± 1
Step	Control	67 ± 5	67 ± 7	40 ± 3	40 ± 2
	ISF	64 ± 6	64 ± 7	39 ± 2	39 ± 2

## Data Availability

The data that support the findings of this study are available upon request from the corresponding author. The data are not publicly available due to privacy or ethical restrictions.
